# Supplementary material for: Induction of inverted morphology in brain organoids by vertical-mixing bioreactors
Source: Commun Biol. 2021 Oct 22;4:1213. doi: 10.1038/s42003-021-02719-5 (PMC8536773; doi:10.1038/s42003-021-02719-5)
Supplement: Supplementary file 2 — Supplementary Information [file 42003_2021_2719_MOESM2_ESM.pdf]

Supplementary Information

Title:

Induction of inverted morphology in brain organoids by vertical-mixing bioreactors

Authors:

Dang Ngoc Anh Suong, Keiko Imamura, Ikuyo Inoue, Ryotaro Kabai, Satoko Sakamoto, Tatsuya Okumura, Yoshikazu Kato, Takayuki Kondo, Yuichiro Yada, William L. Klein, Akira Watanabe, Haruhisa Inoue

Supplementary Figure 1. Vertical mixing system

Supplementary Figure 2. Validation of culture condition for inverted brain organoids

Supplementary Figure 3. Immunostaining and analysis of cilium

Supplementary Figure 4. Gene expression by single-cell RNA sequencing

Supplementary Figure 5. Generation of GABAergic neurons and glutamatergic neurons in orbital mixing and vertical mixing.

Supplementary Table 1. Up-regulated genes in SOX2-positive cells from vertical mixing

Supplementary Table 2. Down-regulated genes in SOX2-positive cells from vertical mixing

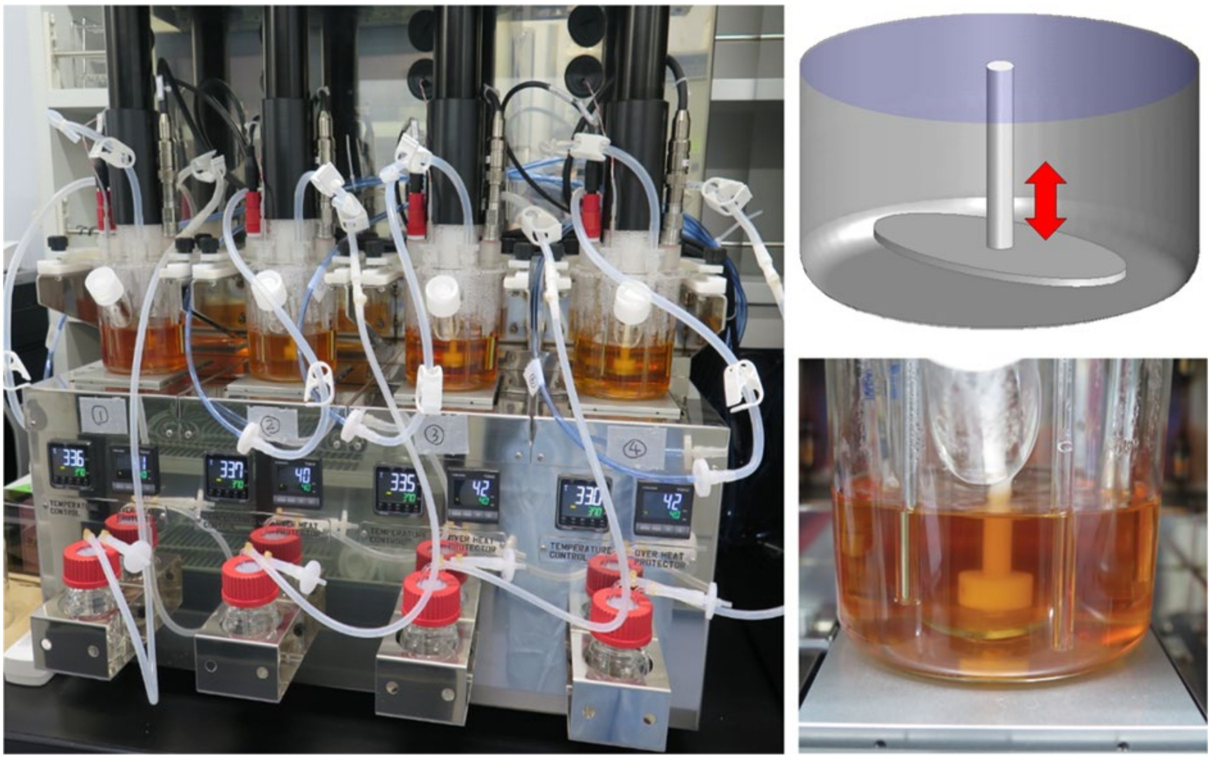

Supplementary Figure 1. Vertical mixing system

The impeller was developed as an unsteady mixing system with vertical motion to create the circulation flow in the culture vessels. The system exhibits 3D dispersion and blending properties with low shear stress. The system can provide an environment for culturing cells that are sensitive to shear.

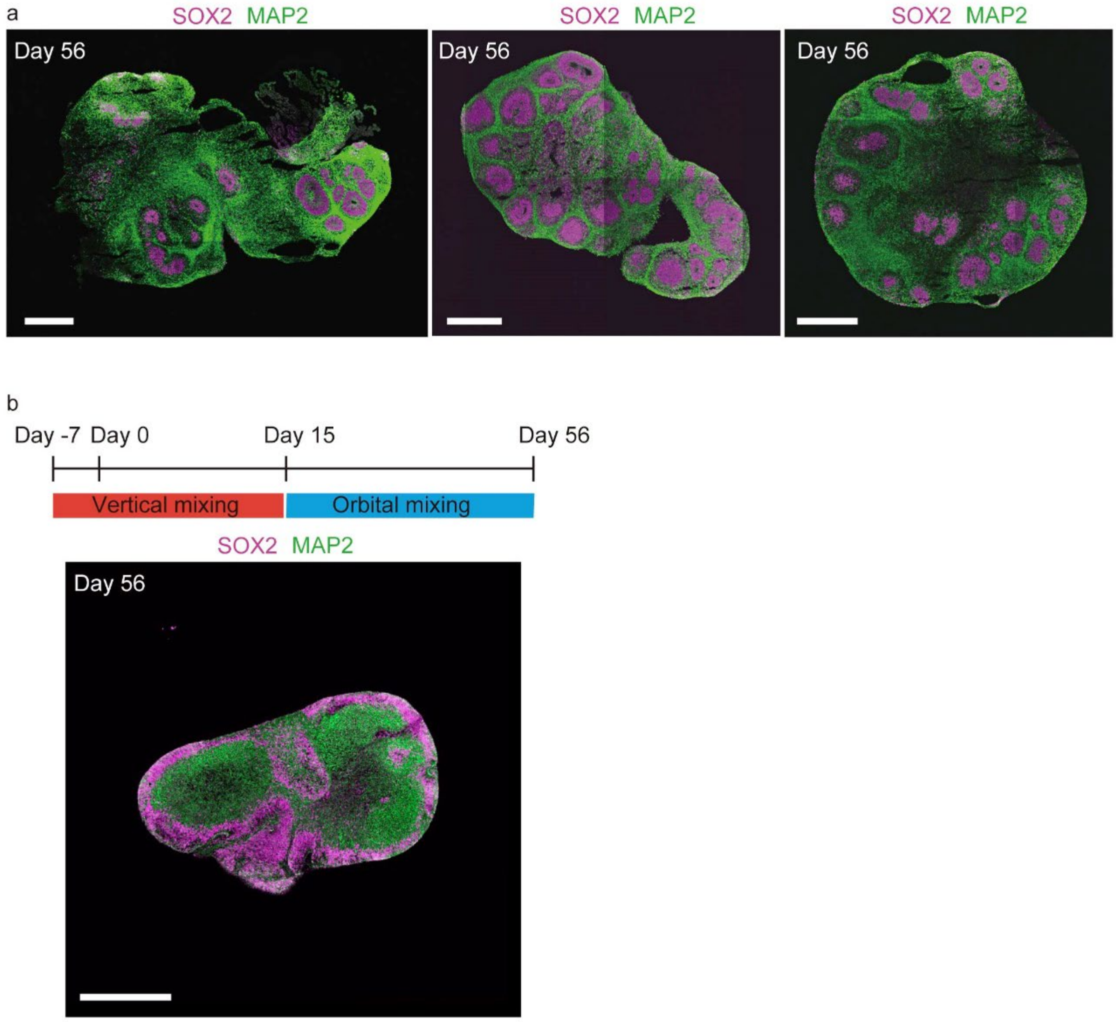

(a) Organoids were generated by orbital mixing without Matrigel. Generated organoids were analyzed by immunostaining with SOX2 and MAP2, and did not present the inverted pattern. Scale bar = 500 µm. The SOX2-positive area in the peripheral region, which is defined as within 100 µm from the edge of brain organoids was 8.4 ± 6.7% (n = 4 organoids, mean ± SD).

(b) Inverted organoids were generated when orbital mixing was initiated on Day 15 following vertical mixing. Scale bar = 500 µm. The % of SOX2-positive area in the peripheral region, defined as within 100 µm from the edge of brain organoids, was 66.0 ± 8.8% (n = 10 organoids, mean ± SD).

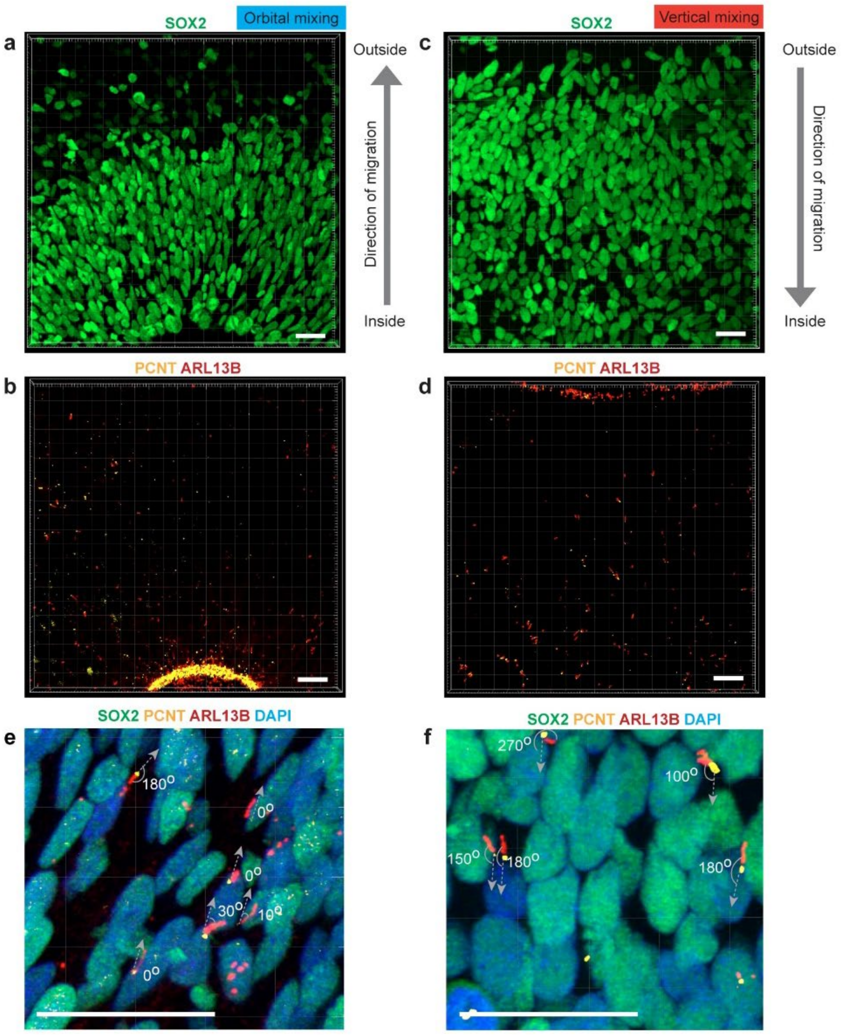

Supplementary Figure 3. Immunostaining and analysis of cilium

(a,c) SOX2 staining, (b,d) PCNT and ARL13B staining, (e,f) measurement of cilia directions. Scale bar = 20 μm.

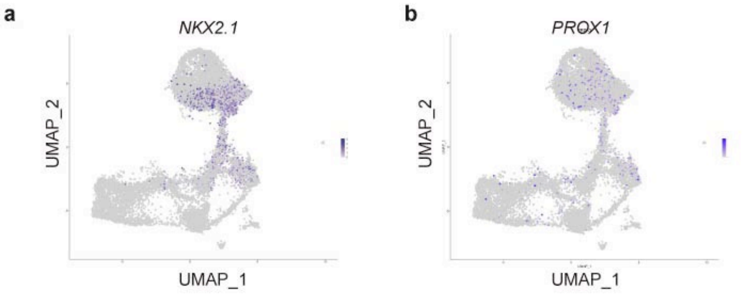

Supplementary Figure 4. Gene expression analysis by single-cell RNA sequencing

Single-cell RNAseq analysis demonstrated that NKX2.1-positive cells (a) and PROX1-positive cells (b) were detected predominantly in organoids generated by vertical mixing.

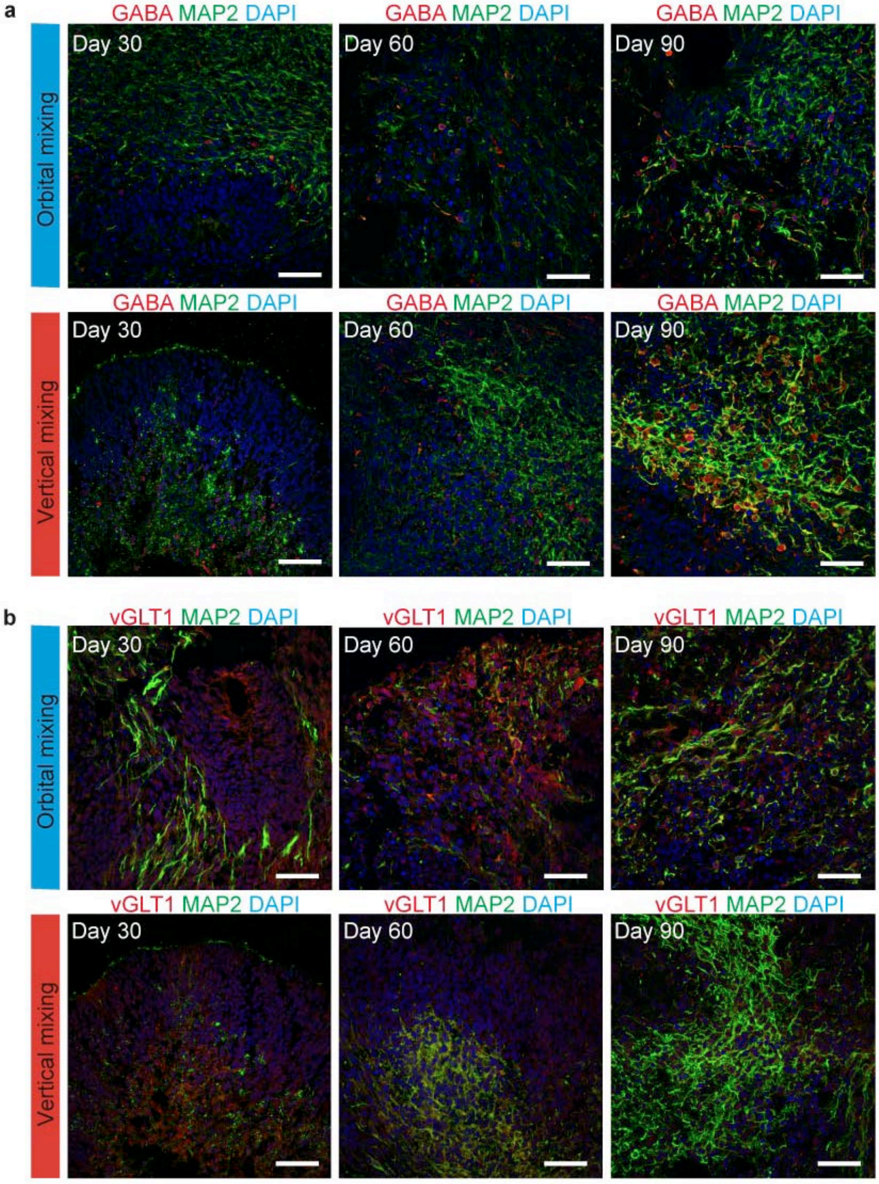

Supplementary Figure 5. Generation of GABAergic neurons and glutamatergic neurons in orbital mixing and vertical mixing.

GABAergic neurons stained with anti-GABA antibody were observed predominantly in organoids by vertical mixing (a), while glutamatergic neurons stained with anti-vGLT1 antibody were observed mostly in organoids by orbital mixing (b). scale bars = 50 μm.

Supplementary Table 1. Up-regulated genes in SOX2-positive cells from vertical mixing

GeneID	Orbital mixing	Vertical mixing	Fold change (vertical mixing / orbital mixing)	p.value	q.value
CCDC88A	0.759778	1.96437341	2.58545709	0	0
MTRNR2L12	2.37591939	3.64688493	1.53493631	0	0
SOX4	2.27050872	3.72438326	1.64032986	0	0
DLX6-AS1	0	2.14468409	-	0	0
DCX	0.90431738	2.5225715	2.78947585	0	0
GAD2	0.00138891	1.03450911	744.836086	2.20E-301	5.08E-297
DLX2	0.00973436	0.93103742	95.6444383	1.10E-295	2.54E-291
ZEB2	0.3541069	1.40115743	3.95687692	7.24E-289	1.68E-284
SOX11	1.30505823	2.53014362	1.93872086	9.27E-263	2.15E-258
DLX5	0.00249197	0.93950452	377.012382	8.58E-261	1.99E-256
GAD1	0.03675392	0.90117058	24.5190321	3.66E-241	8.48E-237
C11orf96	0.05100117	0.85524575	16.7691406	1.40E-231	3.24E-227
DLX1	0.00446442	0.66830383	149.695601	1.26E-216	2.92E-212
SHTN1	0.17017476	1.06233217	6.24259537	5.82E-205	1.35E-200
DCLK2	0.21906521	1.10536684	5.0458346	4.36E-195	1.01E-190
RND3	0.56916175	1.43236161	2.51661607	2.98E-184	6.91E-180
CD24	0.19955072	0.95903316	4.80596185	6.71E-180	1.55E-175
PFN2	0.71909737	1.48504444	2.0651507	3.51E-178	8.12E-174
NRXN3	0.09844675	0.84169354	8.54973431	3.94E-174	9.11E-170
NRXN1	0.3853379	1.11697156	2.89868081	1.82E-158	4.20E-154
STMN2	0.34073129	1.36013482	3.99181074	2.71E-156	6.28E-152
SP9	0.00067514	0.48603813	719.906644	1.11E-150	2.58E-146
ZNF704	0.29047398	1.01001295	3.47712019	8.62E-150	1.99E-145
PLS3	0.19861773	1.07239701	5.39930151	1.38E-149	3.20E-145
DST	0.54597566	1.29870309	2.37868312	1.96E-145	4.54E-141
DLX6	0.00209364	0.47637005	227.532226	3.28E-144	7.59E-140
PDE4DIP	0.24711343	0.99798217	4.03855898	1.21E-139	2.79E-135
MTSS1	0.23608346	0.85876731	3.63755808	1.57E-138	3.63E-134
ERBB4	0.039857	0.77595343	19.468433	7.04E-135	1.63E-130
SNTG1	0.0687308	0.62533505	9.09832391	1.39E-132	3.23E-128

ARL4C	0.46701191	1.1195478	2.39725749	1.94E-131	4.48E-127
TMEM123	0.4922504	1.13110311	2.29782062	5.38E-126	1.24E-121
NKX2.1	0	0.40067581	-	2.56E-119	5.93E-115
DPYSL3	0.6677219	1.28607828	1.92606873	3.97E-119	9.19E-115
SOX6	0.23418299	0.89502795	3.82191695	8.05E-117	1.86E-112
PDZRN3	0.05581664	0.5560509	9.96209835	9.43E-116	2.18E-111
SOX1	0.2177366	0.76037739	3.4921891	3.58E-107	8.29E-103
RIPOR2	0.02255381	0.45594924	20.2160588	1.71E-103	3.95E-99
GAP43	0.52286503	1.10417557	2.11177933	1.79E-102	4.15E-98
MALAT1	5.91126135	6.53790224	1.10600798	1.24E-101	2.88E-97
LSAMP	0.30947076	0.86473301	2.7942317	1.47E-101	3.41E-97
INA	0.09239611	0.55061044	5.95923854	6.34E-101	1.47E-96
NSG2	0.14945988	0.67296957	4.50267697	7.80E-101	1.81E-96
CHD9	0.96569529	1.45122835	1.50278081	4.48E-97	1.04E-92
SCG3	0.21348428	0.68133452	3.19149741	7.58E-95	1.76E-90
DCLK1	0.23852108	0.73304026	3.07327242	1.06E-93	2.45E-89
CRMP1	0.47923243	0.97950424	2.04390223	3.78E-87	8.75E-83
KMT2E	0.93980734	1.39224486	1.48141517	8.74E-87	2.02E-82
ADGRL3	0.04137173	0.39212611	9.47811817	7.48E-86	1.73E-81
TMSB10	2.44426451	2.9512024	1.20739895	1.06E-85	2.46E-81

Supplementary Table 2. Down-regulated genes in SOX2-positive cells from vertical mixing

GeneID	Orbital mixing	Vertical mixing	Fold change (orbital mixing / vertical mixing)	p.value	q.value
DMRTA2	0.39238063	0.00132195	296.818894	0	0
LTBP1	0.37464281	0.02979299	12.5748633	0	0
OTX1	0.23460054	0.00185793	126.269644	0	0
EMX1	0.36999367	0	-	0	0
PTPRG	0.34799504	0.03884472	8.95861975	0	0
FEZF2	0.41123761	0.00339158	121.25266	0	0
BOC	0.40076825	0.02305161	17.3856914	0	0
HES1	1.15669419	0.243326	4.75368098	0	0
NEUROG2	0.31729505	0.00061459	516.27529	0	0
SLC1A3	0.8345205	0.12489669	6.68168614	0	0
PPP2R2B	0.58684752	0.07416828	7.91237861	0	0
NRN1	0.40937578	0.0149235	27.4316179	0	0
ID4	1.49279997	0.25820776	5.78139077	0	0
B3GAT2	0.46982927	0.0642307	7.31471487	0	0
FABP7	2.36366428	0.6266009	3.77220058	0	0
GLI3	0.76533005	0.03752274	20.3964334	0	0
PTN	2.32780456	0.3604745	6.45761225	0	0
COL4A6	0.5197119	0.01775789	29.2665397	0	0
COL4A5	0.51525544	0.05537459	9.30490819	0	0
SOX3	0.53843221	0.01703922	31.599579	0	0
CLU	1.16341148	0.32941384	3.53176259	0	0
SFRP1	1.685252	0.4149081	4.06174768	0	0
PREX2	0.33088221	0.00526177	62.8841782	0	0
LHX2	1.43627172	0.13470217	10.662573	0	0
PAX6	0.9587145	0.07622688	12.5771181	0	0
CCND1	0.3072905	0.01064432	28.8689678	0	0
TENM4	0.3561817	0.04477732	7.95451106	0	0
FAT3	0.37617923	0.02504914	15.0176499	0	0
CDON	0.54039946	0.05450077	9.91544704	0	0

VIM	3.29286101	0.95818504	3.43656065	0	0
FZD8	0.45346486	0.03933707	11.527673	0	0
EMX2OS	0.28601885	0.00669413	42.7268463	0	0
EMX2	1.06146423	0.04181117	25.3870946	0	0
HMGA2	0.33450661	0.00642054	52.0994666	0	0
DACH1	0.67977393	0.03683314	18.4554986	0	0
ZIC2	0.43829053	0.00242318	180.874483	0	0
EFNB2	0.94204244	0.14297288	6.58895916	0	0
KCNG1	0.38450211	0.03093957	12.4275196	0	0
DOK5	0.84192052	0.10167514	8.28049534	0	0
TTYH1	1.25394025	0.29578042	4.23942951	0	0
SVIP	0.49111315	0.08351691	5.88040376	1.32E-306	3.04E-302
GAS1	0.37734408	0.04570669	8.2557742	4.22E-303	9.77E-299
CDH4	0.30983885	0.02180324	14.2106813	1.32E-299	3.05E-295
HMGN3	1.51533811	0.57961551	2.61438503	4.46E-299	1.03E-294
EML1	0.3205862	0.03144953	10.193671	3.58E-297	8.29E-293
CSRP2	0.85543403	0.22885471	3.73789128	5.13E-289	1.19E-284
FOXP1	0.49430594	0.09851846	5.017394	8.59E-289	1.99E-284
NCKAP5	0.33973496	0.03777425	8.99382476	5.31E-284	1.23E-279
GPC3	0.25113445	0.0098977	25.3730035	8.13E-278	1.88E-273
ZIC5	0.15658365	0.00047327	330.854791	6.77E-274	1.57E-269
