# Supplementary material for: Induction of inverted morphology in brain organoids by vertical-mixing bioreactors
Source: Commun Biol. 2021 Oct 22;4:1213. doi: 10.1038/s42003-021-02719-5 (PMC8536773; doi:10.1038/s42003-021-02719-5)
Supplement: Supplementary file 3 — Description of Additional Supplementary Files [file 42003_2021_2719_MOESM3_ESM.pdf]

Description of Additional Supplementary Files

File name: Supplementary Video 1.
Description: Computational simulation of solid-liquid transient analysis by using a discrete phase model to compare the movement of spheres under orbital mixing.

File name: Supplementary Video 2.
Description: Computational simulation of solid-liquid transient analysis by using a discrete phase model to compare the movement of spheres under vertical mixing.

File name: Supplementary Data 1.
Description: The source data of Figure 1d and Figure 2c.
